# The complete mitochondrial genome of *Gyrodactylus gurleyi* (Platyhelminthes: Monogenea)

**DOI:** 10.1080/23802359.2016.1172042

**Published:** 2016-06-20

**Authors:** Hong Zou, Dong Zhang, WenXiang Li, Shun Zhou, ShanGong Wu, GuiTang Wang

**Affiliations:** a Institute of Hydrobiology, Chinese Academy of Sciences, Wuhan, PR China;; b University of Chinese Academy of Sciences, Beijing, PR China

**Keywords:** *Gyrodactylus gurleyi*, Gyrodagtylidae, mitochondrial genome, phylogenetics

## Abstract

*Gyrodactylus gurleyi*, was inhabited on the fins and gills of goldfish (*Carassius auratus*), which belonged to the family Gyrodactylidae. In this study, we sequenced the complete mitochondrial genome of *G. gurleyi* with the total length of 14 771 bp. The mitogenome contained 12 protein-coding genes (PCGs), 22 tRNA genes, two rRNA genes and two major non-coding regions (NC1 and NC2). The overall AT content was 72.1%. In phylogenetic analysis, *G. gurleyi* and *G. kobayashii* clustered together and then united with the clade of other three *Gyrodactylus* species (*G. salaris*, *G. thymalli* and *G. derjavinoides*) with high nodal support.

*Gyrodactylus gurleyi* was collected on the fins and gills of goldfish (*Carassius auratus*) from Wuhan (30°31′23′′N, 114°23′01′′E), China. It was identified by morphology and ITS molecular marker (Li et al. 2013). The specimen (accession no. IHB20150315006) was stored in the Museum of Aquatic Organisms, Institute of Hydrobiology, Chinese Academy of Sciences, Wuhan, China. The sequences of total mt genomic DNA of *G. gurleyi* were retrieved using long PCR and Sanger method of DNA sequencing.

The complete mt genome of *G. gurleyi* was circular and 14 771 bp in size (GenBank accession no. KU659806). It contained 12 protein-coding genes (PCGs, lacking *Atp8*), 22 tRNA genes, two rRNA genes and two major non-coding regions (NC1 and NC2) (Table 1). All the genes were transcribed from the same strand. The nucleotide composition was computed at 29.2%A, 16.9% G, 42.9% T and 11.1% C. The AT content of 72.1% was lower than that of *Paragyrodactylus variegatus* (76.3%), but higher than that of other four *Gyrodactylus* species (*G. salaris*, 62.3%; *G. thymalli*, 62.8%; *G. derjavinoides*, 68.2%; *G. kobayashii*, 71.6%) (Huyse et al. 2007; Plaisance et al. 2007; Huyse et al. 2008; Ye et al. 2014; Zhang et al. 2016).

**Table 1.** Organization of the mitochondrial genome of *Gyrodactylus gurleyi*.

	Position			Intergenic	Codon		
Gene/region	From	To	Size	nucleotides	Start	Stop	Anti-codon
*Cox3*	1	639	639		ATG	TAA	
*tRNA-His*	643	707	65	3			GTG
*Cytb*	711	1784	1074	3	ATG	TAA	
*Nad4l*	1784	2032	249	−1	ATG	TAA	
*Nad4*	2005	3213	1209	−28	ATG	TAA	
*tRNA-Phe*	3216	3281	66	2			GAA
NC1	3282	4064	783				
*Atp6*	4065	4577	513		ATG	TAA	
*Nad2*	4587	5444	858	9	ATG	TAA	
*tRNA-Val*	5449	5513	65	4			TAC
*tRNA-Ala*	5514	5581	68				TGC
*tRNA-Asp*	5584	5648	65	2			GTC
*Nad1*	5649	6536	888		ATG	TAA	
*tRNA-Asn*	6538	6603	66	1			GTT
*tRNA-Pro*	6604	6667	64				TGG
*tRNA-Ile*	6663	6729	67	−5			GAT
*tRNA-Lys*	6730	6793	64				CTT
*Nad3*	6797	7144	348	3	ATG	TAG	
*tRNA-Ser^(AGN)^*(S1)	7145	7203	59				GCT
*tRNA-Trp*	7207	7271	65	3			TCA
*Cox1*	7276	8823	1548	4	ATG	TAA	
*tRNA-Thr*	8836	8900	65	12			TGT
*rrnL*	8900	9853	954	−1			
*tRNA-Cys*	9854	9914	61				GCA
*rrnS*	9915	10,624	710				
*Cox2*	10,625	11,206	582		ATG	TAA	
*tRNA-Glu*	11,319	11,389	71	112			TTC
*Nad6*	11,393	11,875	483	3	ATG	TAG	
*tRNA-Tyr*	11,884	11,951	68	8			GTA
*tRNA-Leu^(CUN)^*(L1)	11,954	12,019	66	2			TAG
*tRNA-Gln*	12,027	12,089	63	7			TTG
*tRNA-Met*	12,091	12,157	67	1			CAT
NC2	12,158	12,940	783				
*tRNA-Ser^(UCN)^*(S2)	12,941	12,998	58				TGA
*tRNA-Leu^(UUR)^*(L2)	12,999	13,066	68				TAA
*tRNA-Arg*	13,071	13,141	71	4			TCG
*Nad5*	13,140	14,690	1551	−2	ATG	TAG	
*tRNA-Gly*	14,702	14,768	67	11			TCC
	14,772	14,771		3			

The length of 12 PCGs was 9942 bp, accounting for 67.3% of the full length of the genome. As the four *Gyrodactylus* species (*G. salaris*, *G. thymalli*, *G. derjavinoides* and *G. kobayashii*), ATG was the unique start codon. The stop codon TAG was only found in three PCGs (*Nad3*, *Nad5* and *Nad6*), whereas TAA in the rest of the PCGs. The size of the 22 tRNA genes was 1374 bp, varying from 58 bp (*tRNA^Ser(AGN)^*) to 71 bp (*tRNA^Glu^*). Nineteen of them had conventional secondary structure, while *tRNA^Ser(AGN)^*, *tRNA^Ser(UCN)^* and *tRNA^Cys^*lacked DHU arms, which was similar to the other known Gyrodactylidae species. The *rrnL* and *rrnS* were 954 bp and 710 bp in size, respectively. They were adjacent to *tRNA^Thr^* (upstream) and *Cox2* (downstream), and separated by *tRNA^Cys^*, as described with other reported monopisthocotyleans (Huyse et al. 2007; Plaisance et al. 2007; Huyse et al. 2008; Perkins et al. 2010; Ye et al. 2014; Zhang et al. 2014a, 2014b, 2016). In addition, there were 37 bp overlapping sequences and 1763 space sequences, among which the biggest were *NC1* (783 bp) and *NC2* (783 bp).

Phylogenetic relationships between *G. gurleyi* and other nine monopisthocotyleans were inferred by using concatenated amino acid sequences of the 12 PCGs. The same tree topology was obtained by two different computational algorithms: Bayesian inference (BI) and maximum likelihood (ML), in which *G. gurleyi* and *G. kobayashii* clustered together and then united with the clade of 3 *Gyrodactylus* species (*G. salaris*, *G. thymalli* and *G. derjavinoides*) with high nodal support (Figure 1). In addition, although *G. gurleyi* and *G. kobayashii* generally parasitized on the same host, sequence alignments showed that 99% (14 623 bp) mitogenome sequence of *G. gurleyi* was covered by that of *G. kobayashii* with only 80% identity. In contrast, *G. salaris* from Atlantic salmon and *G. thymalli* from grayling share 98% identity with 100% mitogenome alignment coverage.

**Figure 1.**
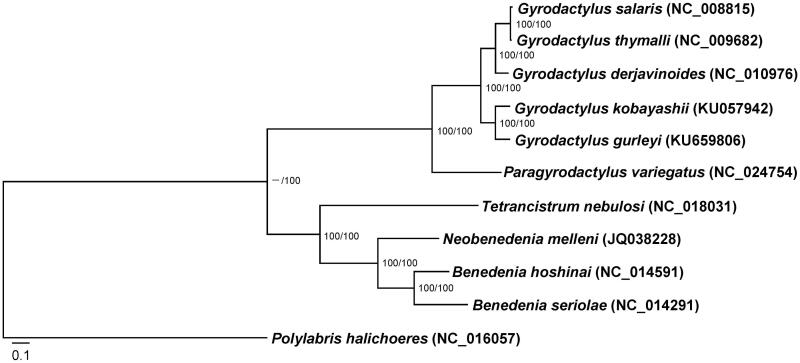
Phylogenetic relationships between *Gyrodactylus gurleyi* and other 9 monopisthocotyleans based on 3044 concatenated amino acid sequences representing 12 mitochondrial protein-coding genes, with *Polylabris halichoeres* used as an outgroup. The MtZoa model for maximum-likelihood analysis and MtREV model for Bayes analysis are selected. Scale bar corresponds to the estimated number of substitutions per site. Bootstrap support values in percent units above nodes are displayed as follows: maximum likelihood bootstrap/Bayesian posterior probabilities.
